# Enzyme Immobilization on Nanoporous Gold: A Review

**DOI:** 10.1177/1178626417748607

**Published:** 2017-12-17

**Authors:** Keith J Stine

**Affiliations:** Department of Chemistry and Biochemistry, Center for Nanoscience, University of Missouri–St. Louis, St. Louis, MO, USA

**Keywords:** Enzyme, immobilization, nanoporous gold, biosensor, biofuel cell, stabilization

## Abstract

Nanoporous gold (referred to as np-Au or NPG) has emerged over the past 10 years as a new support for enzyme immobilization. The material has appealing features of ease of preparation, tunability of pore size, high surface to volume ratio, and compatibility with multiple strategies for enzyme immobilization. The np-Au material is especially of interest for immobilization of redox enzymes for biosensor and biofuel cell applications given the ability to construct electrodes of high surface area and stability. Adjustment of the pore size of np-Au can yield enhancements in enzyme thermal stability. Glucose oxidase immobilization on np-Au has been a focus for development of glucose sensors. Immobilization of laccase and related enzymes has demonstrated the utility of np-Au for construction of biofuel cells. Np-Au has been used to immobilize other redox enzymes, enzyme conjugates for use in bioassays, and enzymes of interest for industrial processes.

## Introduction

Enzyme immobilization is important for a number of applications including the development of biosensors and assays,^1^ of enzyme reactors for industrial processes,^2^ and of use of supported enzymes for biotransformations and enzyme-directed synthesis,^3^ among others. Enzyme immobilization strategies include physisorption, entrapment, cross-linking, and formation of a covalent bond to the support. Physisorption is simple to perform but the enzyme will be subjected to a greater possibility of leaching out of the material than the other methods. Covalent bonding to the support prevents leaching but is subject to enzyme orientation and coverage-related effects. Entrapment proves to be fairly simple and provides good enzyme activity and stability. Nanoporous gold (referred to here as np-Au, but also referred to as NPG in many places) is a newer support with the unique property of being a stable and highly conductive support ideal for use as an electrode. Although it cannot be directly subjected to bioconjugation reactions, it can be modified with self-assembled monolayers (SAMs) presenting terminal functional groups onto which bioconjugation reactions can be used to immobilize enzymes. Np-Au has also proven suitable for physisorption of enzymes, entrapment, and electrostatic immobilization. The studies to date have focused almost exclusively on immobilization on np-Au of oxidoreductases and hydrolases. Examples of all of these modes of enzyme immobilization onto np-Au will be covered during this review.

### Properties, preparation, and characterization of nanoporous gold

**Figure 1.**
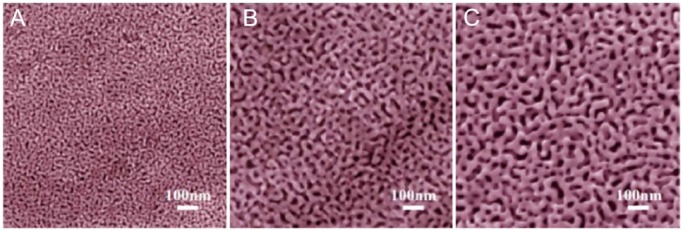
Scanning electron microscopic images of np-Au with pore sizes of (A) 18 nm, (B) 30 nm, and (C) 50 nm. The scale bar in each image is 100 nm. Reproduced with permission from Chen et al,^9^ copyright Elsevier.

The structure of np-Au consists of interconnected ligaments with gaps between the ligaments that define pores of irregular morphology, as shown in Figure 1.^4^ The size of the pores can be varied from less than 10 nm to several hundred nanometers by adjusting the preparation method. Np-Au provides a high surface area, usefulness as an electrode, and capacity to be surface modified with SAMs of alkanethiolates and related species. The np-Au material can be prepared in a variety of formats, including free-standing structures,^5^ supported thin films,^6^ and thin sheets affixed to electrode surfaces.^7^ Surface modification and application of np-Au in analytical chemistry have been reviewed.^8^

Thus far, the most common approaches to preparation of np-Au start with alloys of Au and a less noble metal such as Ag,^10^ Cu,^11^ Sn,^12^ or Zn^13^ with the atomic percentage of Au between 20% and 50%. Treatment with strong acid can remove the less noble element in a process referred to as dealloying and result in np-Au. If the Au composition is less than 20%, then dealloying will result in the alloy falling apart, whereas an Au composition above 50% will not give a nanoporous structure and will instead yield separated pits. Dealloying in electrolyte solution can be achieved using the precursor alloy as an electrode and applying a potential positive enough to oxidize the less noble metal.^14,15^ The use of an applied electrochemical potential gives control over the final pore size. Increasing the potential at which dealloying occurs was found to give a steadily smaller pore size, decreasing from 16 to 9 nm as the potential was increased from 0.8 to 1.3 V.^16^ Dealloying at low temperature in strong acid can be used to obtain smaller size pores of 7 nm on dealloying at −20°C and 15 nm on dealloying at 0°C.^17^ Alloys of Au with Al can be dealloyed to form np-Au by treatment with concentrated sodium hydroxide solution.^18^ The mechanism of the dealloying process has been described as a combination of diffusion of the less noble metal into solution and surface diffusion and reorganization of the remaining Au atoms into random interconnected ligament structures.^19^

There are also methods for preparing np-Au directly by etching of an Au electrode. One method reported involved anodizing Au at 1.8 V in a solution containing sodium oxalate, resulting in a 1-μm-thick film with pore size near 20 nm.^20^ A np-Au film was produced by anodization at 2.0 V in sulfuric acid solution, yielding an average pore size of 32 nm.^21^ Etching of Au sputter coated onto Si wafers by anodization in a 1:1 v/v solution of aqueous hydrofluoric acid and dimethylformamide was used to produce np-Au films.^22^ Potential cycling in an electrolyte of zinc chloride and benzyl alcohol for 30 cycles produced a 5-μm-thick np-Au film with pores in the range 30 to 200 nm depending on the cycle times.^23^ In this strategy, Zn is deposited on the cathodic scan and dealloying occurs on the anodic scan.

Control over np-Au pore size is important for a number of reasons. The pores must be of adequate size to allow an enzyme to diffuse inside the material. It has been predicted that a pore dimension of 2 to 6 times the dimensions of the enzyme is optimal for improving enzyme stability.^24^ After preparation of np-Au, the pore size may be increased either by thermal annealing or by electrochemical treatment. The annealing of np-Au and the increase in pore size depend on the surface diffusivity of Au atoms which increases with temperature.^25^ Exposing a sample of np-Au to a fixed higher temperature below the melting point of Au for a fixed period of time will promote coarsening and give larger pores and wider ligaments.^26^ A method to adjust the pore size of np-Au by applying square wave potential waveforms in hydrochloric acid solution was introduced.^27^ In this method, above a critical potential, chloroaurate anions form at the gold surface and diffuse away into solution. In a related study, application of cyclic waveforms to np-Au in different electrolyte solutions resulted in coarsening and also to np-Au for which the pore sizes and ligament thicknesses were quite different from each other.^28^

The specific surface area of np-Au free-standing structures of sufficient mass, generally at least 100 mg, may be determined using nitrogen gas adsorption isotherms and the BET (Brunauer, Emmett, and Teller) analysis of the gas adsorption isotherm.^25^ The pore size distribution can be determined by the BJH (Barrett, Joyner, and Halenda) analysis. For np-Au electrodes, surface area can be determined using cyclic voltammetry by an anodic scan to produce a monolayer of gold oxide that is then reduced in the cathodic scan.^28^ Examination of the np-Au by scanning electron microscopy together with image analysis can provide information on pore size distribution. Electron tomography has been used to provide 3-dimensional morphology maps of np-Au.^29^

### Immobilization of glucose oxidase

Given the high interest in glucose detection for the management of diabetes, immobilization of glucose oxidase (GOx) is of crucial importance to development of electrochemical biosensors. Electrochemical sensors based on immobilization of GOx continue to be the major commercial approach for the monitoring of glucose in diabetes management and progress has been reviewed.^30^ Immobilization of GOx on nanomaterials for sensor development is an emerging field^31^ and hence immobilization on np-Au is also being actively pursued. Glucose oxidase is a 160-kDa dimeric flavoenzyme that uses flavin adenine dinucleotide (FAD) as a redox cofactor. Flavin adenine dinucleotide is noncovalently bound near the active sites of the enzyme. Glucose oxidase catalyzes the oxidation of β-d-glucose to d-glucono-1,5-lactone which is then hydrolyzed to gluconic acid. During the enzyme cycle, FAD is reduced to the hydroquinone form FADH_2_ by accepting 2 electrons and 2 protons. Flavin adenine dinucleotide can be regenerated from FADH_2_ by oxidation by molecular oxygen, mediators, or in some instances by direct electron transfer. The regeneration of FAD from FADH_2_ generates H_2_O_2_ which can be detected electrochemically. An important feature of np-Au in this detection is the finding that hydrogen peroxide (H_2_O_2_) can be oxidized at a lower potential on np-Au than on flat gold, a phenomenon attributed to the higher number of steps on the np-Au material.^32^ Oxidation of H_2_O_2_ at lower potentials decreases the possibility of interference in serum samples from species such as ascorbic acid or uric acid. Direct oxidation of β-d-glucose on np-Au electrodes has also been reported.^33–35^

Np-Au prepared from 100-nm-thick Au/Ag alloy was affixed to a glassy carbon electrode and enzyme solution was coated and allowed to dry.^32^ A coating of Nafion was applied to prevent enzyme leakage. The resulting GOx enzyme electrode was used at a potential of +0.4 V (vs saturated calomel electrode [SCE]) to detect H_2_O_2_ during the oxidation of glucose. A linear range from 1 to 18 mM glucose concentration was found with a detection limit of 196 μM and a sensitivity of 0.049 μA mM^−1^. After 1 month of storage at 4°C, the electrode lost 4.2% of its current response. Lignin peroxidase, useful for degrading aromatic pollutants, is an enzyme that is dependent on H_2_O_2_ for its activity. Lignin peroxidase was co-immobilized on np-Au with GOx by physisorption.^36^ Decolorization of 3 dyes by the co-immobilized enzymes with H_2_O_2_ supplied to lignin peroxidase by action of GOx on glucose was successfully observed. Np-Au nanowire arrays were created inside alumina templates followed by etching away the alumina and used to immobilize GOx.^37^ The reduction in H_2_O_2_ from activity of immobilized GOx was detected at −0.1 V (vs Ag/AgCl) and a linear range from 0.05 to 2 mM glucose concentration was observed with a detection limit of 0.046 mM. The electrode retained 85% of its response after 7 days of measurements and no interference from the presence of ascorbic acid was observed.

**Figure 2.**
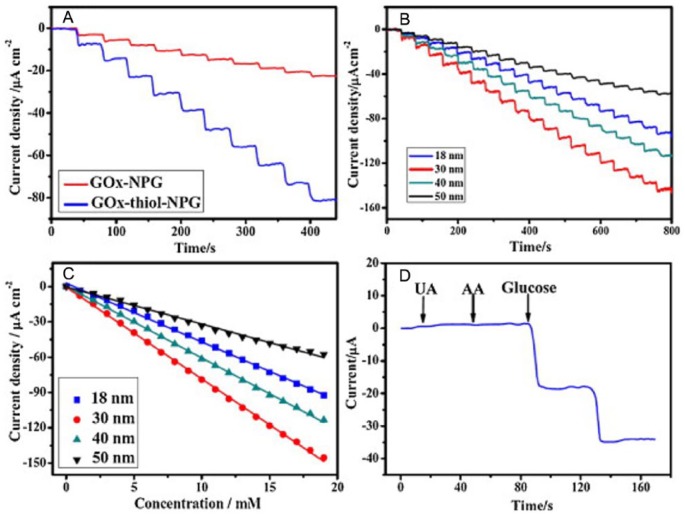
(A) Current-time curves of 2 GOx-modified np-Au samples with pore size of 30 nm for successive addition of 1 mM glucose in 0.1 M PBS at −0.2 V. (B) Chronoamperometry curves and (C) calibration curves of np-Au with different pore sizes in 0.1 M PBS and KCl solution with successive addition of 1 mM glucose at potential of −0.2 V. (D) Chronoamperometry curve of np-Au with pore size of 30 nm in 0.1 M PBS and KCl solution with successive addition of 0.02 mM UA, 0.1 mM AA, and 1 mM glucose. AA indicates ascorbic acid; GOx, glucose oxidase; NPG, nanoporous gold; PBS, phosphate-buffered saline; UA, uric acid. Reproduced with permission from Chen et al,^9^ copyright Elsevier.

Covalent attachment of GOx to surface-modified np-Au has been used to make a glucose sensor.^9^ Alloy leaves of composition Au_35_Ag_65_ were treated with nitric acid and then modified with dithiobis (succinimidyl undecanoate) to form an activated SAM on the Au surface ready for reaction with amines of lysines on GOx. The modified np-Au was affixed to a glassy carbon electrode. The pore size was controlled by varying the dealloying time from 15 minutes (18 nm) to 8 hours (50 nm). Detection of H_2_O_2_ at −0.2 V (vs Ag/AgCl) was most sensitive for the electrode of smallest pore size. A linear range for response to glucose was found that extended up to near 20 mM with a detection limit of 10 μM. No response was observed for the interferents uric acid or ascorbic acid. The highest current response to glucose was found for np-Au with pore size 30 nm. Figure 2 shows the variation of current density for successive additions of glucose vs time and concentration and confirmation that under the conditions noted uric acid and ascorbic acid did not interfere with the measurements.

**Figure 3.**
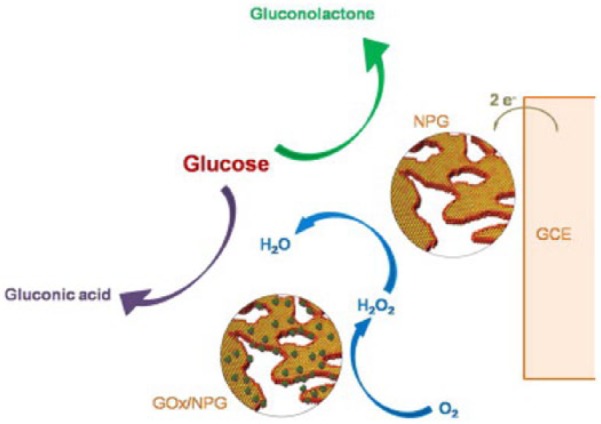
Electrochemical reaction on the surface of the GOx/np-Au/GCE bioelectrode. GCE indicates glassy carbon electrode; GOx, glucose oxidase; NPG, nanoporous gold. Reproduced with permission from Wu et al,^38^ copyright Elsevier.

The observation of nonenzymatic catalysis of glucose oxidation by bare np-Au motivated a study to see whether glucose oxidation on GOx-modified np-Au could proceed by both enzymatic and nonenzymatic routes at the same time.^38^ Glucose oxidase was immobilized by physisorption onto np-Au formed by dealloying 12-carat gold leaf and affixing it to a glassy carbon electrode. An illustration of the reaction occurring on the enzyme-modified np-Au surface is shown in Figure 3. In deaerated solution, nonenzymatic oxidation of glucose was observed on GOx-modified np-Au. At a potential of 0.3 V (vs SCE), current from both enzymatic and nonenzymatic glucose oxidation was measured. A linear range from 0.050 to 10 mM glucose was observed, with a detection limit of 1.02 μM and negligible effect of interferents. As a part of this study, glucose levels were determined in 4 human serum samples using the modified np-Au electrode and agreement to within ~5% or better with the results from an automatic biochemical analyzer was found. This report is one of the few so far concerning clinical applications of an enzyme-modified np-Au electrode.

Mediated electron transfer between np-Au and GOx has also been used to create a glucose sensor.^39^ Np-Au thin films, created by dealloying Au + Ag alloys co-sputtered onto glass supports with a 10-nm titanium adhesion layer, were modified by GOx, a bifunctional cross-linker and an osmium-containing redox polymer. The mixture was applied to the np-Au electrode as a drop and allowed to dry. The structure of the modified electrode is one of the pores penetrated by the GOx-containing hydrogel with mediated electron transfer from the GOx cofactors and the osmium-containing redox polymers. A detection limit of 2.0 μM glucose was found in cyclic voltammetry sweeps for the modified np-Au electrode with the current density still increasing at 50 mM glucose.

Glucose oxidase has also been entrapped in np-Au modified by conducting polymer.^40^ Np-Au of 30-nm pore size was prepared by dealloying 12-carat gold leaf and then affixing it to a glassy carbon electrode. A solution of 3,4-ethylenedioxythiophene monomer, polyethylene glycol of molecular weight 20 000, and GOx was polymerized by cyclic voltammetry sweeps creating the poly(3,4-ethylenedioxythiophene) film inside np-Au entrapping the enzyme. The detection of H_2_O_2_ was performed at 0.200 V (vs SCE). A wide linear range from 0.1 to 15.0 mM glucose was reported and noted as covering the relevant range in blood of 2 to 10 mM. A detection limit of 10 μM was reported.

The performance of GOx on np-Au for sensing glucose in the presence of mediators in solutions that were N_2_ (g) degassed was studied.^41^ The mediators *p*-benzoquinone and ferrocene carboxylic acid were used to shuttle electrons from the FAD/FADH_2_ cofactors in GOx to the np-Au surface. The np-Au electrode was prepared by dealloying 100-nm-thick 12-carat Au sheets and affixing them to glassy carbon electrodes. The np-Au was modified by an SAM of cysteamine, treated with glutaraldehyde, and then GOx was covalently bound. The ferrocene carboxylic acid mediator behaved similarly on flat Au and on np-Au but *p*-benzoquinone showed a different behavior on np-Au than on flat Au, with narrower peak separation and negatively shifted peaks in cyclic voltammograms. The response to glucose was tested at 0.2 V (vs SCE) for *p*-benzoquinone and at 0.3 V for ferrocene carboxylic acid. A linear response up to at least 10 mM was observed, and the response of the *p*-benzoquinone mediator was more sensitive. Similar np-Au electrodes were surface modified with SAMs of 3,3′-dithiopropionic acid, mercaptohexanoic acid, or mercaptoundecanoic acid and then GOx was covalently linked to the SAMs by *N*-(3-dimethylaminopropyl)-*N*′-ethylcarbodiimide/*N*-hydroxysuccinimide (EDC/NHS) coupling chemistry.^42^ Glucose was detected in deaerated solutions using the *p*-benzoquinone mediator. It was found that the peak separation widened and the peak current decreased for *p*-benzoquinone as the chain length of the SAM increased.

**Figure 4.**
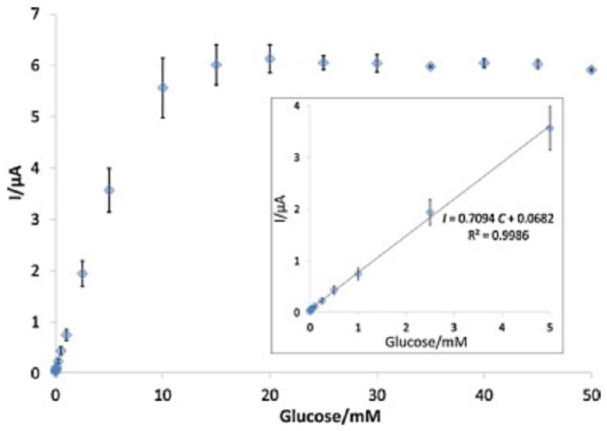
Amperometric tests (at 0.52 V vs SCE) of the GOx_ads_-hPG electrode in the presence of various glucose concentrations in the electrolyte (range of 5-50 mM). Error bars refer to 3 replicates. hPG indicates highly porous gold; SCE, saturated calomel electrode. Reproduced with permission from du Toit et al,^43^ copyright Elsevier.

A material referred to as “highly porous gold” (hPG) was created and used for GOx immobilization.^43^ The material is formed by electrodeposition of Au onto Au disc electrodes from a solution of HAuCl_4_ and NH_4_Cl at a highly negative potential of −4.0 V (vs SCE) where hydrogen bubbles form. The hPG film has a combination of large pores in the microns range lined by nanopores. It was found that immobilizing GOx by cyclic voltammetry scans from 0.42 to 0.60 V (vs SCE) gave superior adsorption and greater activity than simple adsorption from solution, a phenomenon attributed to electrostatic interaction between the Au surface and negatively charged enzyme. The modified electrode was used to detect glucose covering a linear range up to 10 mM and with a detection limit of 25 μM. The *K_m_* value for the immobilized enzyme was 6.3 mM compared with 27 mM for GOx in solution. A Michaelis-Menten–like plot for current vs glucose concentration was found, as shown in Figure 4. A material prepared in a similar manner and referred to as “nanocorral Au” was used for GOx immobilization by cross-linking with glutaraldehyde.^44^ The material had an open cell foam–like morphology on the microns scale and a corral-like appearance on the nanometers scale. A linear range for glucose detection from 0.005 to 3.0 mM was found, and the *K_m_* for the immobilized GOx was 3.2 mM. The immobilized GOx was about twice as sensitive to glucose as GOx similarly immobilized on flat Au.

### Immobilization of laccase and bilirubin oxidase

Laccase and bilirubin oxidase (BOD) are multicopper oxidase proteins,^45^ and these enzymes are key to the development of enzymatic biofuel cells that have the prospect of functioning in physiological media and powering implantable devices.^46^ It is necessary to develop their immobilization on materials suitable for use as cathodes in biofuel cells. Given its tunable porosity and utility as an electrode, np-Au is of high interest for biofuel cell development. These enzymes contain 4 copper ions at 3 types of copper centers: T1, T2, and T3. There are 2 T3 sites. An electron from the substrate is transferred to site T1 which is then transferred intramolecularly through a histidine-cysteine-histidine (His-Cys-His) bridge to a T2/T3 trinuclear copper center where O_2_ is reduced to H_2_O. Laccase^47^ and BOD^48^ are able to oxidize a large variety of substrates.

**Figure 5.**
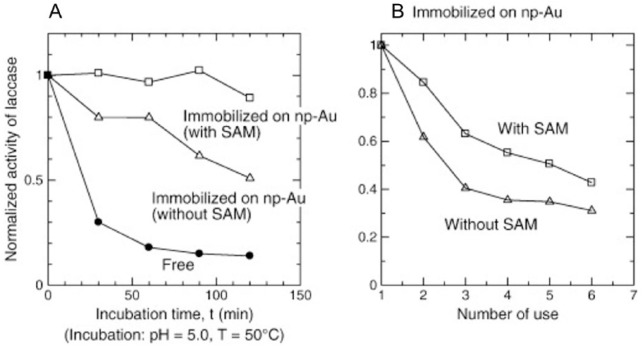
Effect of (A) incubation time and (B) reuse number, on enzyme activity for free and immobilized laccase. Activities shown on the vertical axes were normalized to initial activity. SAM indicates self-assembled monolayer. Reproduced with permission from Hakamada et al,^52^ copyright Elsevier.

Laccase was immobilized on np-Au by physisorption from solution.^49^ Np-Au was prepared from Au/Ag alloy sheets (50:50 wt%) 25-μm thick or 100-nm thick. The amount of laccase immobilized was determined using the Bradford assay. Laccase activity was monitored at 470 nm using 2,6-dimethoxyphenol as the substrate. Np-Au of pore size 40 to 50 nm was used. Np-Au of pore size 10 to 20 nm accommodated much less enzyme explained as due to the smaller pore size blocking diffusion of laccase into the material. The immobilized enzyme showed much greater thermal stability than free enzyme, retaining 60% of its activity at 50°C after 2 hours as compared with only 6% retained activity for the free enzyme. Cyclic voltammograms measured on enzyme-loaded electrodes of the 100-nm np-Au placed on top of glassy carbon showed clear electrocatalytic reduction in dissolved oxygen. In a subsequent study, 3 modes of immobilization of laccase onto np-Au were compared: covalent, electrostatic, and physisorption.^50^ The amount of laccase loaded onto np-Au was almost the same for covalent attachment and physisorption. Covalent attachment was achieved by modifying np-Au with an SAM of lipoic acid, forming active esters by treatment with EDC/NHS chemistry, and reaction with surface lysine residues on the enzyme. Electrostatic immobilization was achieved by first modifying np-Au with methylene blue to provide a positive surface as the isoelectric point of laccase is 3.4. Although electrostatic immobilization resulted in loading about half as much enzyme, the activity of enzyme achieved was similar by all 3 methods. A particle size effect was observed, with enzyme on smaller microns sized np-Au fragments having slightly lower *K_m_* and slightly higher *k_cat_* values. Direct immobilization of laccase onto np-Au modified by an SAM of 4-aminothiophenol was found to give cyclic voltammograms in which peaks associated with the T1 and T2 metal centers could be distinguished.^51^ An enzyme stabilization effect was observed on np-Au with the peak current increasing with number of cycles. A biofuel cell was then constructed using np-Au modified with 4-aminothiophenol and laccase as the cathode and np-Au modified with 4-aminothiophenol and GOx as the anode. Glucose was used as the fuel, being oxidized at the anode, and O_2_ was reduced to H_2_O at the cathode. Immobilization onto either np-Au or 4-aminothiophenol–modified np-Au was found to improve the thermal stability of laccase.^52^ On incubation at 50°C for 30 minutes, free laccase lost 70% of its activity, whereas laccase immobilized on np-Au lost only 20% and laccase immobilized on 4-aminothiophenol–modified np-Au lost only 10% of its activity after 120 minutes incubation (see Figure 5).

Np-Au electrodes were modified by placing a drop of BOD solution under vacuum, allowing it to dry, and then placing a drop of a copolymer epoxy over the electrode.^53^ The addition of the epoxy significant stabilized the electrocatalytic current of the immobilized BOD for oxygen reduction over multiple cycles. A study of immobilization of BOD on np-Au thin films of varying pore size and thickness showed higher enzyme loading and current densities for BOD immobilized on np-Au of smaller pore size.^54^ The highest current density was found for pores of size 8 ± 2 nm, too large to allow enzyme penetration, and thus the high current density was attributed to surface adsorption. The np-Au was made by magnetron sputtering onto glass slides of a 10-nm titanium adhesion layer followed by Au and Ag of composition Au_30_Ag_70_. Dealloying time and temperature were varied to yield a range of pore sizes. The BOD was drop cast in solution onto the np-Au electrodes, dried under vacuum, and then sealed with poly(ethylene glycol) diglycidyl ether. A subsequent study sought to optimize np-Au thickness and pore size for use of BOD together with glucose dehydrogenase (GDH) in biofuel cells.^55^ The BOD was covalently attached to np-Au modified by aminophenyl groups and then the remainder of the Au surface was blocked by mercaptopropionic acid. For the BOD cathodes, the highest current density of 800 μA cm^−2^ was found for 500-nm-thick np-Au films with 17-nm pore size. For the GDH anodes, the highest current density was found for 300-nm-thick np-Au films of 16-nm pore size. The resulting biofuel cell gave a power density of 17.5 μW cm^−2^ at 0.193 V in 50 mM phosphate buffer with 5 mM glucose. In artificial serum, the power density was 7.0 μW cm^−2^ at 0.166 V. The presence of cracks in the np-Au films was noted as playing an important role in facilitating substrate access to the enzymes. In a subsequent study, a supercapacitor/biofuel cell hybrid device was made using GDH on np-Au as the anode and BOD on np-Au as the cathode, with the ability to operate more than 50 charge/discharge cycles.^56^

**Figure 6.**
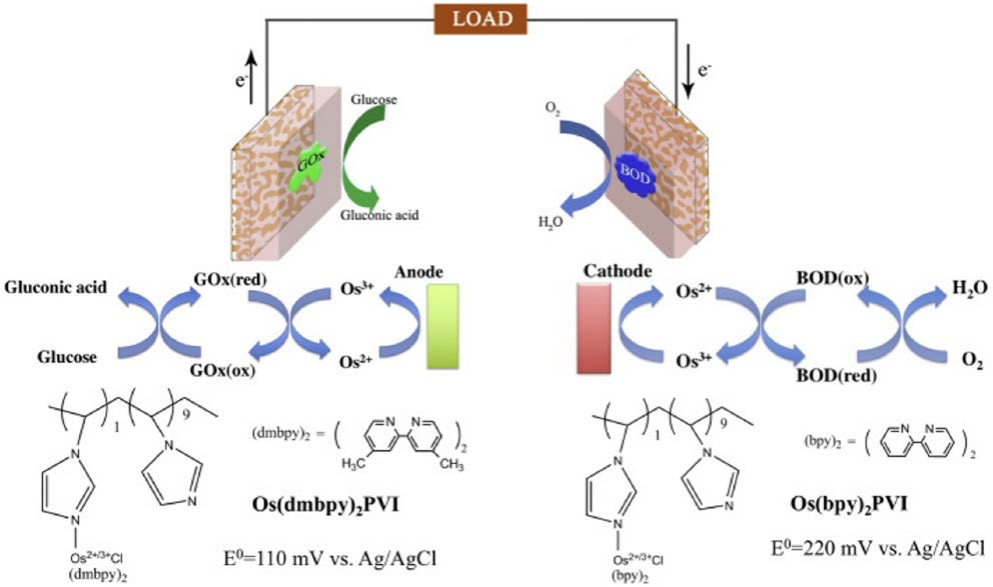
Schematic diagram of an np-Au–based glucose/O_2_ biofuel cell fabricated by drop casting a solution of osmium redox polymer, enzyme, and cross-linkers. BOD indicates bilirubin oxidase; GOx, glucose oxidase. Reproduced with permission from Xiao et al,^58^ copyright Elsevier.

Laccase or BOD immobilized on np-Au have been used together with the enzyme cellobiose dehydrogenase (CDH) immobilized on np-Au to create biofuel cells.^39^ Cellobiose dehydrogenase oxidizes cellobiose to cellobio-δ-lactone but can also use lactose or glucose as a substrate. In the reported approach, all enzymes were mixed with osmium-containing redox polymer and poly(ethylene glycol) diglycidyl ether before being drop cast onto np-Au electrodes and allowed to dry. A CDH-modified electrode was found to have a limit of detection of 6.0 ± 0.4 μM for lactose and 16.0 ± 0.1 μM for glucose. The CDH-modified np-Au was used as the anode with either laccase or BOD-modified np-Au as the cathode in biofuel cells. The biofuel cell with CDH as the anode and BOD as the cathode gave a power density of 41 μW cm^−2^ in 5 mM lactose, and the biofuel cell with laccase as the cathode gave a power density of 24 μW cm^−2^ in 5 mM lactose. Biofuel cells based on np-Au electrodes were studied in nonaqueous solvents.^57^ A diagram of the components of this biofuel cell is shown in Figure 6.^58^ The enzymes BOD and GOx were immobilized on np-Au together with osmium-containing redox polymers and poly(ethylene glycol) diglycidyl ether. In O_2_-bubbled phosphate-buffered saline with 5 mM glucose, an open-circuit voltage of 0.56 V and a power density of 3.65 μW cm^−2^ at 0.21 V were reported. In 95% acetonitrile, the power density was reduced to 0.47 μW cm^−2^ and the open-circuit voltage shifted to 0.36 V. In a series of alcohols from methanol to pentanol, the power density decreased with increasing hydrophobicity of the alcohol.

A biofuel cell constructed from hPG electrodes and using laccase and GOx was reported.^43^ Glucose oxidase was adsorbed onto hPG under conditions of potential scanning and laccase was adsorbed onto hPG that had been surface modified electrochemically with aminophenyl groups by first reacting *p*-nitrophenyldiazonium tetrafluoroborate with the Au surface under cathodic conditions and then reducing the nitro groups to amines. In an aerated buffer containing 27.8 mM glucose, a power density of 6 μW cm^−2^ at 0.2 V was achieved. There is a strong interest in constructing flow-through enzymatic biofuel cells for use as implanted devices. Using hPG electrodes deposited onto Pt wires and modified with laccase and GOx, flow-through biofuel cells were constructed in 2 configurations using 3-dimensional–printed molds.^59^ Glucose oxidase was electrochemically adsorbed and laccase was attached to the aminophenyl-modified hPG following treatment with 6-mercapto-1-hexanol and EDC/NHS. A single flow-through channel containing the cathode and anode as repeatedly folded Pt wires was compared with a bifurcated flow channel design with straight Pt electrodes for the cathode and anode. The flow-through biofuel cells were fed with 27 mM glucose in aerated solution at 0.35 mL min^−1^. The design with 2 channels gave a higher power density. The overall power output was fairly steady from both designs over 24 hours, with a higher power output from the single channel design. The power output from the flow cells decayed significantly over a period of a month.

Laccase has previously been immobilized onto carbon nanotubes and inside redox polymer matrices for the construction of biofuel cell cathodes.^60^ Biofuel cells constructed in this manner have overall higher power densities than most of those reported for the enzyme immobilized in np-Au with values as high as 12 mW cm^−2^ reported for pulsed discharges. Evaluation of laccase immobilized on np-Au of greater thickness for the biocathode may result in greater power densities. However, the power densities achieved to date are compatible with the requirements for wearable devices.^61^

### Immobilization of other enzymes

Investigation of a wider range of enzymes on np-Au for various applications has begun to be reported, including both redox and other types of enzymes acting on a wider variety of substrates.

Acetylcholinesterase is of high interest in sensors for detection of organophosphorous compounds such as pesticides^62^ and nerve agents.^63^ The enzyme was immobilized onto np-Au by physisorption onto samples of differing average pore size (50-100 nm vs 200 nm) prepared by treating 2.0 cm × 2.0 cm plates of a 10-carat white gold foil (41.8 atomic% gold) of 250 μm thickness in nitric acid for either 24 or 72 hours.^64^ Enzyme kinetics was studied using the Ellman assay with acetylthiocholine as the substrate. The value of *K_m_* was found to increase from 0.08 mM in solution to 0.26 mM for the enzyme on the 50 to 100 nm pore size np-Au and to 0.15 mM for the enzyme on the 200 nm pore size np-Au. The enzyme loading was determined by analyzing the reduction in the activity of the supernatant compared with the initial solution. Submonolayer coverages of enzyme were found, dependent on the concentration of the bulk enzyme solution. Using measured *V_max_* values and estimated enzyme loading, it was determined that *k_cat_* for acetylcholinesterase was reduced from the observed value in solution on adsorption onto np-Au.

Np-Au modified with multiwalled carbon nanotubes (MWCNT) was used for the immobilization of acetylcholinesterase to create a sensor for organophosphates using the pesticide malathion as a test substrate.^65^ In this case, the np-Au was prepared by a repetitive alloying/dealloying process applied to a gold electrode by electrochemical cycling in ZnCl_2_ dissolved in benzyl alcohol. Multiwalled carbon nanotubes were shortened by reflux in HNO_3_, and carboxylic acid groups were introduced at each end. The np-Au was then surface modified with cysteamine and exposed to a solution of MWCNTs. After immobilization of the MWCNTs, the np-Au was exposed to a solution of acetylcholinesterase by drop casting and drying. Action of the enzyme on the substrate acetylthiocholine produced thiocholine whose oxidation at the Au surface can be detected amperometrically. The enzyme electrode in the presence of this substrate generated a large catalytic current peak near 913 mV (vs SCE). Inhibition of the peak current by malathion gave a detection limit of 0.5 ng mL^−1^ and a linear range from 1 to 500 ng mL^−1^.

Immobilized lipases are important in the production of biofuels and other applications.^66^ Immobilization of lipase on np-Au was used to make an electrochemical sensor for triglycerides in human serum.^67^ The enzyme was physisorbed on an electrode prepared by attachment of dealloyed 12-carat Au leaf onto a glassy carbon electrode. Substrates of tributyrin, olive oil, and human serum samples were tested. The hydrolysis of the triglycerides released protons whose reduction was detected by electron transfer through gold to glassy carbon. Use of the electrode gave values for the amount of triglycerides in 3 serum samples in better than 5% agreement with results from an automatic biochemical analyzer. This study is another of the very few so far applying enzyme-modified np-Au in clinical analysis. Immobilization of porcine pancreatic lipase on np-Au of pore sizes 25 and 47 nm was studied.^68^ Covalent linkage of enzyme to the Au surface through lipoic acid using EDC/NHS and physical adsorption were compared. The enzyme loading into np-Au was similar for covalent attachment and physical adsorption. However, the stability to storage over a period of 30 days was very good for covalently immobilized enzyme, whereas adsorbed enzyme showed significant loss of activity. The loading of enzyme was less on np-Au of 47 nm pore size. The thermal stability of immobilized lipase in 25 nm pore size np-Au found to be very good with 93% of the activity retained after incubation at 70°C for 30 minutes in buffer. This stability is comparable or superior to reports for thermal stability of porcine pancreatic lipase on other material supports. For example, porcine pancreatic lipase immobilized on short rod-shaped mesoporous silica retained about 50% of its activity after 1 hour of incubation at 50°C.^69^ Porcine pancreatic lipase immobilized on mesoporous silica modified by 3-aminopropyltrimethoxysilane was reported to retain about 50% of its activity after incubation at 60°C (pH 7.0) for 1 hour.^70^ The results for lipase immobilization on np-Au suggest that good and possibly improved thermal stabilization can be achieved and may depend on pore size optimization.

Lignin peroxidase was immobilized on np-Au of 40 to 50 nm average pore diameter by physisorption.^35^ The immobilized enzyme lost about 20% of its activity in 1 hour at 45°C compared with free enzyme which lost almost all its activity. The immobilized enzyme was found to be effective at decolorization of dye molecules including fuchsine, rhodamine B, and pyrogallol red, meant to demonstrate that the immobilized enzyme could be useful for degrading aromatic pollutants in water. The immobilized enzyme retained 95% of its activity when stored at 4°C for a month.

The enzyme xylanase, useful for hemicellulose conversion in industrial processes, was immobilized by physisorption onto np-Au formed by dealloying 25-μm-thick Au_42_Ag_58_ alloy foil.^71^ X-ray photoelectron spectroscopy data indicated a role for formation of Au-S bonds involving cysteine residues. The activity of the immobilized enzyme was studied using xylan as the substrate and determining the amount of xylose produced by stopping the reaction with 3,5-dintrosalicylic acid, which reacts with reducing sugars, and measuring the absorbance at 550 nm. Immobilization increased *K_m_* from 0.12 to 0.27 mM, and decreased *k_cat_* from 4024 to 3539 min^−1^.

For the dual applications of removal of Pb^2+^ and degradation of toxic di(ethylhexyl)phthalate from drinking water, the enzyme cutinase was immobilized on np-Au that was first surface modified with polyethyleneimine (20 000 average molecular weight).^72^ The np-Au was surface modified with lipoic acid and then treated with chloroacetylchlorine in pyridine/chloroform to activate the surface followed by exposure to the polymer in dimethylformamide. Cutinase was then physically adsorbed and its activity confirmed by hydrolysis of *p*-nitrophenyl butyrate by observing the *p*-nitrophenol product by its absorbance at 405 nm. The enzyme and polymer-loaded np-Au was effective at removing Pb^2+^ from drinking water and at degrading di(ethylhexyl)phthalate by hydrolysis to 1,3-isobenzofurandione, a nontoxic by-product.

Np-Au prepared by dealloying 25-μm-thick alloy foil of composition Au_22_Ag_78_ was used to immobilize lipase from *Pseudomonas cepacia*, catalase, and horseradish peroxidase by physisorption.^73^ The enzyme loading was determined by analyzing the supernatant and collected washings of the enzyme-loaded np-Au using the Bradford assay. The np-Au prepared had an average pore size of 35 nm. Enzyme activity was studied using *p*-nitrophenyl palmitate as the substrate for lipase and pyrogallol as the substrate for horseradish peroxidase. The activity of catalase was assayed indirectly by stopping the reaction with ammonium molybdate which forms a complex with hydrogen peroxide. Conversion of soybean oil to biodiesel was demonstrated using the immobilized lipase. Immobilization of lipase on np-Au improved its thermal stability, with 76% of the enzyme activity retained after incubation at 70°C for 30 minutes vs retention of 54% of activity for free lipase. X-ray photoelectron spectroscopy data gave evidence for Au-S and Au-N interaction which could result in strong attachment of the enzymes to the Au surface after simple physisorption. In a subsequent study, this lab found that the leaching of lipase from np-Au could be minimized by proper choice of pore size with no leaching observed for 35 nm pore size np-Au after 10 cycles of use.^74^ The inhibition of horseradish peroxidase oxidation of the substrate *o*-phenylenediamine by sulfide ions was used to create a sensor for sulfide concentration.^75^ The horseradish peroxidase enzyme was drop cast from solution onto np-Au. The current associated with reduction in oxidized *o*-phenylenediamine at the Au surface was measured over a range of sulfide concentration. The range of detection of the sensor was 0.1 to 40 mM and the limit of detection was 0.027 μM. Horseradish peroxidase immobilized on np-Au/glassy carbon electrodes was found active for oxidation of *p*-aminophenol, *o*-phenylenediamine, *p*-phenylenediamine, and catechol.^76^ The enzyme was immobilized by physisorption. Wide linear ranges and low μM detection limits were observed for detection by differential pulse voltammetry. Distinctly separated oxidation peaks were observed for *p*-aminophenol, *o*-phenylenediamine, and *p*-phenylenediamine. An electrochemical immunoassay for hepatitis B antigen was designed using an np-Au electrode and gold nanoparticles.^77^ The np-Au electrode was modified with 3,3′-dithiopropionic acid di(*N*-succinimidyl ester) that was then conjugated to antibody. The Au nanoparticles were modified with horseradish peroxidase and a second antibody. Formation of the immunocomplex on np-Au in the presence of antigen was detected by the action of the enzyme on *o*-phenylenediamine with detection of the product by differential pulse voltammetry.

Np-Au has been used as a support for an immunoassay for prostate-specific antigen (PSA) based on the immobilization of a monoclonal antibody conjugated to the enzyme alkaline phosphatase.^78^ Np-Au was prepared as an approximately 10-μm-thick coating on a gold wire by electrodeposition of a gold and silver alloy of 20 atomic% gold followed by dealloying in nitric acid. The antibody-enzyme conjugate was linked by the EDC coupling reaction to an SAM of lipoic acid formed on the NPG. This immunoassay was based on the principle of inhibition of enzyme activity on antigen binding with enzyme activity assessed using *p*-nitrophenyl phosphate as the substrate and spectrophotometric detection of *p*-nitrophenolate product at 410 nm. The assay was found to respond linearly to PSA up to 20 ng mL^−1^ with a detection limit of 0.1 ng mL^−1^.

Np-Au prepared on Au wire electrodes was also modified with SAMs of lipoic acid and used to create electrochemical biosensors for biomarkers PSA and carcinoembryonic antigen (CEA).^79^ Conjugates of IgG monoclonal antibodies with alkaline phosphatase were covalently linked to the lipoic acid SAMs by EDC/NHS coupling. The production of *p*-aminophenol product from *p*-aminophenylphosphate substrate was determined using the square wave voltammetry peak current for the oxidation of *p*-aminophenol to *p*-quinoneimine near 0.2 V (vs Ag/AgCl). Binding of the PSA or CEA antigen resulted in a reduction in the peak current that was proportional to the antigen concentration. The linear range for PSA response extended up to 30 ng mL^−1^ and for CEA extended up to 10 ng mL^−1^. The detection limit for PSA was 0.75 ng mL^−1^ and was 0.015 ng mL^−1^ for CEA. Similarly prepared np-Au electrodes were used to immobilize conjugates of lectin concanavalin A and alkaline phosphatase onto lipoic acid SAMs.^80^ Using square wave voltammetry and *p*-aminophenylphosphate substrate, responses to binding of the glycoproteins transferrin or IgG were measured through the decrease in the peak current associated with substrate conversion. Competitive assays were achieved using immobilized glycoprotein and competition between glycoprotein and lectin-enzyme conjugate in solution for binding to the surface.

The enzyme fructose dehydrogenase (FDH), a 140-kDa flavoenzyme, was immobilized on np-Au with the pore size varied between 9 and 62 nm by variation of the dealloying time and temperature.^81^ Fructose dehydrogenase–based biosensors have applications in food analysis.^82,83^ The np-Au was in one case covalently modified with mercaptopropionic acid. The np-Au was also modified electrochemically by 2-carboxy-6-naphthoyl diazonium salt which was reduced onto the surface to form a covalent bond. The enzyme was allowed to physisorb onto the surface and then EDC was added to promote amide bond formation. The maximum in catalytic current density was found for a pore size of 42 nm, large enough to allow good penetration of enzyme into the material but not too large so that loading would decrease. The immobilized enzyme activity was stable after 6 days of storage at 25% of the initial activity on mercaptopropionic acid–modified np-Au and at 40% of the initial activity on np-Au modified by the diazonium salt. The catalytic current density was maximal at 35°C and decreased at higher temperatures. The enzyme electrodes responded to fructose over the range of 0.05 to 0.3 mM with a detection limit of 1.2 μM. Good agreement was found for fructose concentration in a series of food samples as measured by the FDH enzyme electrode with that determined by a commercial kit for fructose determination. Alcohol dehydrogenase was loaded onto np-Au sheets of 100 nm thickness that were affixed to glassy carbon electrodes.^31^ The enzyme was applied by drop casting in solution followed by sealing the electrode with Nafion. Biosensing of ethanol was conducted at 0.5 V (vs SCE) by detecting the oxidation of nicotinamide adenine dinucleotide cofactor. The detection limit was 120 μM and the linear range extended to 8 mM.

## Conclusions

Np-Au has emerged as a substrate of major importance for enzyme immobilization. The advantages of stability, biocompatibility, and availability of many immobilization strategies will encourage additional applications of np-Au in the development of enzyme-based biosensors, biofuel cells, and supported enzyme catalysis for industrial and synthetic applications. Enzyme immobilization on other matrices such as mesoporous silica materials,^84^ carbon nanotube nanomaterials,^85,86^ polymer resin particles,^87^ fibrous materials,^88^ polymer gels,^89^ and nanoparticles^90^ has been previously studied. Np-Au is a worthy addition to the range of materials for enzyme immobilization, and some comparisons that point out some favorable features of np-Au are presented in Table 1. Np-Au offers some advantageous features in terms of stability in virtually all relevant environments, intrinsic conductivity, ease of fabrication in a variety of formats, and availability of multiple strategies for enzyme immobilization.

**Table 1.** Comparison of some of the advantages and disadvantages of a selection of matrices for enzyme immobilization.

Matrix	Advantages	Disadvantages
Nanoporous Au	Stable in wide range of pH Variability of format Wide range of accessible pore size Electrical conductivity	Annealing of pore size with time Self-assembled monolayer instability Cost
Mesoporous silica	Uniform pore diameter High surface area Good for electrostatic immobilization Direct modification for covalent immobilization	pH sensitivity
Sol-gel	Suitable for enzyme entrapment	Brittle pH sensitivity Substrate diffusion limitations
Carbon nanotubes	Conductivity High surface area	Challenging functionalization Fragility Aggregation
Porous polymer	Preparation inside molded shapes Preparation as polymer beads	Complex chemistry Separation from product mixture Substrate diffusion limitations

Although most studies of immobilization on np-Au have focused on oxidoreductases and hydrolases, other kinds of enzymes could also be suitable for immobilization such as lyases and isomerases as well as RNA-based or DNA-based enzymes, and future studies along these directions can be expected. Np-Au can be prepared with large enough pore size such that DNA-editing enzymes could be entrapped and DNA sequences passed through np-Au using flow techniques.^91^ Another area remaining open for exploration is that of immobilization of multienzyme complexes that perform sequential catalytic transformations. Np-Au is suitable for integration into microfluidics^92^ and into flow chemistry arrangements^93^ for applications in flow-through enzyme reactors for small-scale applications such as the production of small quantities of biologically significant oligosaccharides.

A major future direction for application of enzyme-modified np-Au should be the analysis of clinical samples, for measurement of metabolites, hormones, and small molecule biomarkers for health and disease. A potential advantage of the adjustable pore size of np-Au is the use of pore sizes that will exclude some larger species that might foul an electrode but allow entrance of smaller molecules for detection. Development of enzyme-linked immunoassays with electrochemical detection applied to clinical samples is also a promising avenue. The types of clinical samples to be explored include serum, urine, tears, and others. Np-Au is suitable for fabrication in electrode array format,^94^ and electrode arrays of enzyme-modified np-Au may find applications in clinical analysis. Continued development of enzyme-modified np-Au toward use in wearable or implantable devices powered by biofuel cells is likely given the stability and biocompatibility of np-Au.
